# Precariousness and depressed mood: a network analysis in the multi-ethnic HELIUS study

**DOI:** 10.1007/s00127-026-03072-w

**Published:** 2026-03-13

**Authors:** Leonie K. Elsenburg, Karien Stronks, Henrike Galenkamp, Jeroen Lakerveld, Anja Lok, Kyuri Park, Vítor V. Vasconcelos, Mary Nicolaou

**Affiliations:** 1 Department of Public and Occupational Health, Amsterdam UMC, Amsterdam Public Health Research Institute, University of Amsterdam, Amsterdam, The Netherlands; 2 The Centre for Urban Mental Health, University of Amsterdam, Amsterdam, the Netherlands; 3 Department of Epidemiology & Data Science, Amsterdam Public Health Research Institute, Amsterdam UMC, Vrije Universiteit Amsterdam, Amsterdam, the Netherlands; 4 Upstream Team, Amsterdam UMC, Vrije Universiteit Amsterdam, Amsterdam, the Netherlands; 5 Department of Psychiatry, Amsterdam UMC, Location AMC, Amsterdam, the Netherlands; 6 Computational Science Lab, Informatics Institute, University of Amsterdam, Amsterdam, the Netherlands; 7 POLDER center, Institute for Advanced Study, University of Amsterdam, Amsterdam, the Netherlands

**Keywords:** Precariousness, Depression, Depressed mood, Ethnic origin, Network analysis

## Abstract

**Purpose:**

Precariousness, which refers to experiencing a high level of insecurity and instability in life, manifests in multiple life dimensions and can give rise to mental health issues. Associations with mental health are potentially influenced by migration background. In this study, we examine the associations between precariousness in different life dimensions and depressed mood among individuals with and without migration background.

**Methods:**

We included 22,039 participants from the baseline measurement of the HELIUS (HEalthy LIfe in an Urban Setting) study, representing the six largest ethnic groups in Amsterdam. We used 13 indicators of precariousness in five life dimensions (employment, financial, housing, cultural and social), 9 assessed through a self-report questionnaire and 4 from neighborhood-level data. Depressed mood was classified as > 9 on the Patient Health Questionnaire (PHQ-9, Dutch version). Network models were applied, stratified by migration background.

**Results:**

In total, 14.6% experienced depressed mood. Among those who experienced precariousness in at least four dimensions, the corresponding number was 22.1% to 46%, depending on the specific dimensions of precariousness that were experienced. Associations between depressed mood and indicators of precariousness were similar for those with and without a migration background. Depressed mood was associated with marginal work or unemployment, social satisfaction, social frequency, income inadequacy, discrimination, and financial difficulties, and additionally with lost friendship and health literacy among those with a migration background.

**Conclusion:**

Individuals with and without depressed mood differ markedly in their experience of precariousness, but associations are mostly consistent between those with and without a migration background.

**Supplementary Information:**

The online version contains supplementary material available at https://doi.org/10.1007/s00127-026-03072-w.

## Introduction

Precariousness is a multi-dimensional concept describing a high level of insecurity and instability in people’s lives. Precarious can manifest in different dimensions of life. Dimensions of life in which this insecurity and instability can manifest are employment, finances, housing, culture and social relations [1]. For employment, this instability and insecurity can take the form of being fired, being excluded from the labor market or experiencing marginal employment. Having an unstable income, financial issues or having trouble to make ends meet creates instability and insecurity in people’s financial situation. For housing, living in an unsafe neighborhood or a neighborhood with low resources can be considered to create insecurity in people’s lives. Insecurity or instability in people’s lives can also relate to feeling excluded from society through experiencing discrimination or not being able to participate fully due to low literacy. Finally, having few social relations or feeling socially excluded can create insecurity and instability in people’s lives. Individually, these experiences are not likely to result in a state of precariousness, but combined they are. Importantly, precariousness experiences can include both objective conditions and subjective experiences [1]. While the concept of precariousness fits within the tradition of research on social determinants of health, it is restricted to conditions and experiences describing insecurity or instability in people’s lives.

The experience of insecurity and instability in the different life dimensions are related and are important for physical and mental health, including depression [2–5]. Precariousness and depressed mood may be linked through multiple mechanisms, including limited access to resources, exposure to adverse psychosocial environments, chronic stress, maladaptive health behaviors, inadequate social support, restricted healthcare access, and heightened feelings of inferiority, injustice, and powerlessness [4, 6, 7].

Previous studies have shown associations between experiences of precariousness and mental health and self-reported health. Precarious employment, and more specific indicators such as job insecurity or temporary employment, were shown to have an adverse effect on mental health [8, 9]. Recently, it was found that, rather than having a non-permanent job, the perception of being in a precarious situation impacted young people’s mental health [10]. In Europe, transitioning into housing arrears, i.e. not being able to pay for the household’s main dwelling on time, was associated with a deterioration in self-reported health, even when factors such as educational level, marital status, presence of a chronic illness, income, and job loss were taken into account [3]. The association was stronger among renters compared with homeowners with mortgages, with renters already starting from a lower baseline of health. Additionally, reductions in housing benefits for low-income individuals renting in the private sector in the UK led to an increase in depressive symptoms [11].

This highlights how deeply interconnected these adverse conditions and experiences are, and how they relate to health. However, existing studies have yet to establish whether all forms of precariousness are associated with depressed mood, or which specific dimensions contribute most to the development of depression. This gap in knowledge is particularly critical for designing effective prevention strategies, as identifying the most influential risk factors could help target interventions more precisely and mitigate the mental health consequences of precarious living conditions.

In this study, we examine the associations between precariousness and depressed mood in a multi-ethnic population. In a previous study, we demonstrated that the interrelationship between precariousness indicators varies across those with and those without a migration background [12]. Moreover, both the prevalence of indicators of precariousness — such as income level [13] and working conditions [14] — as well as the prevalence of depressive symptoms [15] have been shown to be unevenly distributed among those with and those without a migration background. Therefore, the associations between precariousness indicators and depressed mood may also differ between those with and without a migration background. In this study, we will explore the association between precariousness indicators and depressed mood in a multi-ethnic population, stratified by migration background. In line with the widely accepted Dutch standard, both immigrants and their offspring are considered to have a migration background [16].

## Methods

We used data from the multi-ethnic HELIUS (HEalthy LIfe in an Urban Setting) study at baseline [13, 14, 17]. These data were enriched with neighborhood exposure data from various sources, provided by the Geoscience and Health Cohort Consortium (GECCO) [18, 19].

The HELIUS study was approved by the Medical Ethics Committee of the Academic Medical Center (AMC, current Amsterdam UMC). Stratified by ethnic origin group, subjects were randomly selected from the Amsterdam municipality register to obtain representative samples of each ethnic origin group. Stratified sampling was performed to allow for equal sample sizes across the six largest ethnic origin groups in Amsterdam to be included. These groups migrated to the Netherlands between the 1960s and the 1990s. Potential participants received a written invitation and a reminder after 2 weeks. Of those who did not respond to the written invitation, 35% were visited by an interviewer of the same ethnic origin group (24% of which were not contacted after five visits). Of those invited, 55% were contacted by written invitation or through a home visit. The percentage contacted in the different ethnic origin groups ranged between 46 and 62%. Of those contacted, 50% agreed to participate with percentages in the different ethnic origin groups ranging between 41 and 61%. The overall response rate was 28% and 24,780 participants were included in the study at baseline (2011–2015). Participants received the questionnaire (either digitally or on paper, depending on their preference) and a letter confirming the appointment for the physical examination. Help with completing the questionnaire was offered when necessary by a trained same-sex interviewer of the same ethnic origin group who spoke their preferred language. In total, 23,936 participants filled in the questionnaire. Written informed consent was obtained from all participants. Details about recruitment into and representativeness of the study sample can be found in Snijder et al. (2017) [13].

### Precariousness and mental health indicators

Table 1 lists the precariousness indicators across the different dimensions and the mental health indicators included in the current study. The precariousness indicators describe a state of insecurity and instability that can be experienced in the various life dimensions and relate to having work or being fired (‘employment’), financial situation (‘financial’), neighborhood characteristics (‘housing’), cultural experiences and a sense of belonging (‘cultural’), and interpersonal relationships (‘social’). All included indicators were dichotomized (see Table 1). Of the indicators, 11 were extracted from the baseline questionnaire of HELIUS and 4 were derived from the neighborhood-level data of GECCO (i.e., the housing indicators and the indicator ‘low number of cultural locations’).

**Table 1:** Dichotomization of the precariousness and mental health indicators

Dimension	Operationalization of indicators
Employment	1. Marginal work or unemployment
	No: Paid work for ≥ 12 h/pension/full-time homemaker/in education,
	Yes: Paid work for < 12 h/unemployed/unfit for work (incapacitated)/public assistance
	2. Fired or became unemployed < 12 months
	No: No, Yes: Yes
Financial	1. Income inadequacy (‘In the past year, have you struggled to make ends meet with your household income?’)
	No: No, not at all/No, but I do have to pay attention to my expenses, Yes: Yes, a little trouble/Yes, a lot of trouble
	2. Serious financial difficulties < 12 months
	No: No, Yes: Yes
Housing^a^	1. Low safety score neighborhood^a^
	No: The upper 75%, Yes: The lowest 25%
	2. Low area resources score neighborhood^a^
	No: The upper 75%, Yes: The lowest 25%
	3. High percentage rental properties neighborhood^a^
	No: The lowest 75%, Yes: The upper 25%
Cultural	1. Perceived discrimination – measured using the Dutch adaptation of the Everyday Discrimination Scale (9 items) [20, 21]
	No: Never/hardly ever/not so often on all items, Yes: pretty often/often on at least one item
	2. Low health literacy – measured using the Dutch adaptation of Chew’s Set of Brief Screening Questions (SBSQ) [22-24]
	No: mean score > 3, Yes: mean score of ≤ 3
	Range of the mean score: 1–5 with 5 considered high health literacy
	3. Low number of cultural locations neighborhood^a^
	No: The upper 75%, Yes: The lowest 25%
Social	1. Lost a friendship, issue with a friend or break-up < 12 months^b^
	No: No, Yes: Yes
	2. Low frequency social support (transactions) – measured using the Daily Emotional Support Subscale of the Social Support Questionnaire for Transactions (SSQT) [25]
	No: sum score ≥ 15, Yes: sum score < 15
	Range of the sum score: 5–20 and 20 considered high frequency of social support [26]
	3. Low adequacy social support (satisfaction) measured via the Daily Emotional Support Subscale of the Social Support Questionnaire for Satisfaction with the supportive transactions (SSQS) [27]
	No: sum score ≥ 15, Yes: sum score < 15
	Range of the sum score: 5–20 and 20 considered high satisfaction with social support [26]
Health	1. Depressed mood (high depressive symptoms)
	No: A score < 10 on the PHQ-9, Yes: A score ≥ 10 on the PHQ-9 (Dutch version of the Patient Health Questionnaire [28])^c, d,e^
	Range of the score: 0–27 and 27 considered high depressive symptoms
	2. Low general mental health
	No: A score > 42 on the MCS-12, Yes: A score ≤ 42 on the MCS-12 (Mental Component Summary of the Short Form Health Survey 12 (SF-12) [29-31]^f, g^
	Range of the score: 0-100 and 0 considered poor general mental health [30]

^a^Based on administrative neighborhood-level geo-data collected as part of the Geoscience and Health Cohort Consortium (GECCO) [18, 19]; ^b^Reporting the occurrence of at least one of these three events results in ‘yes’ and reporting no information on any of the three events results in missing on this indicator; ^c^If one of the items was missing, it was replaced by the mean score of the other items. If more than one item was missing, no sum score was calculated; ^d^Item 8 of the PHQ-9 originally contained two questions and turned out to be very difficult to answer when pre-testing the HELIUS questionnaire. Therefore, item 8 was divided into 2 items in the questionnaire and again combined into a single item before calculating the sum score [32]; ^e^In the HELIUS study, measurement invariance of the PHQ-9 regarding ethnic origin was shown [32]; ^f^In the HELIUS study, the SF-12 did not show measurement invariance regarding ethnic origin [33], ^g^Four items were reverse coded to ensure that higher scores represent better health [33].

### Migration background

To determine the ethnic origin of participants, registered country of birth of participants and of their parents was used, which is the most widely accepted indicator of ethnic origin in the Netherlands [16]. Participants of Dutch, Surinamese, Ghanaian, Turkish, or Moroccan origin were included in the HELIUS study. When a participant was born outside the Netherlands and at least one parent was born outside the Netherlands, a participant was a first-generation migrant and of non-Dutch ethnic origin. When a participant was born in the Netherlands, while both parents were born outside the Netherlands, a participant was a second-generation migrant and of non-Dutch ethnic origin. Individuals were considered of Dutch ethnic origin and to not have a migration background when they were born in the Netherlands and their parents were also born in the Netherlands. Whether Surinamese participants were of South-Asian, African or Javanese origin was determined on their self-report.

### Demographic factors

Age and sex were extracted from the municipal registry. The educational level was based on self-reported highest educational level and divided into four categories. Occupational level was based on participants’ current job title and description (self-reported) and divided into five categories according to the Dutch Standard Occupational Classification system 2010. Participants who indicated not to be in employment reported the title and description of their last job. Marital status was also based on self-reported information and divided into five categories.

### Statistical analysis

Using the dichotomized indicators of precariousness in the employment, financial, housing, cultural, and social dimension (Table 1), a sum score per dimension as well as an overall precariousness score was determined. To make sure that all dimensions could contribute the same score to this precariousness score, the maximum allowed sum score per dimension was 2. Descriptive statistics of demographic factors, the precariousness indicators and the precariousness score are presented according to depressed mood as well as overall. We also created Venn diagrams of the overlap between the experience of precariousness in the five mentioned dimensions (operationalized as any experience in that dimension) and the experience of depressed mood in the study sample. Additionally, we created an overview of the percentage who experienced depressed mood within each of the combinations of dimensions in which precariousness can be experienced.

Secondly, we examined network models of the precariousness indicators and depressed mood. The purpose of the network analysis was to explore the complex interrelations between indicators of precariousness and depressed mood from a systems perspective. Specifically, by applying an Ising model to dichotomized indicators, we aimed to identify which aspects of precariousness are uniquely associated with depressed mood after adjusting for all other indicators in the network. This approach moves beyond simple pairwise associations and enables the identification of direct and indirect statistical dependencies between variables. Moreover, network analysis allows us to visualize and interpret the structure of these relationships, providing insight into how different life dimensions are interrelated in the context of depressed mood. This was done separately for those with and those without a migration background. We applied an Ising model to the dichotomized indicators of depressed mood and precariousness in the employment, financial, housing, cultural, and social dimension using the bootnet package (version 1.6) in R (version 4.3.2) [34]. An Ising model is an undirected weighted network in which the variables are the nodes, and the edges are adjusted standardized regression coefficients which we refer to as the association between the variables (derived through regularized logistic regression). The coefficients are adjusted for all other variables in the network. We used the default hyperparameter of 0.25 and the ‘AND’ rule for obtaining the results. This means that weak edges are considered to be ‘0’ and that for edges to be present, they need to be bidirectional. The network was visualized using the ‘qgraph’ package (version 1.9.8) and the Fruchterman-Reingold algorithm (Epskamp et al., 2012). The accuracy of the models was tested by nonparametric bootstrapping the CIs (*n* = 1,000) [35].

Finally, as a robustness test to our findings regarding associations between precariousness and depressed mood, we performed the second step of this analysis for general mental health instead of depressed mood to examine whether we observed the same the results when using another mental health indicator.

## Results

From this study, we excluded individuals of unknown ethnic origin (*n* = 50) and subsequently those with missing information on one of the 13 precariousness indicators in the employment, financial, housing, cultural, and social dimension (*n* = 1,794). At most, 2.2% of the sample was missing information on any of these indicators. From the main analysis, we also excluded those with more than one missing item on depressed mood (*n* = 53). The final sample for the main analysis was 22,039 individuals (92% of the baseline population). In additional analyses, we excluded those with missing information on general mental health (*n* = 144), instead of those with missing information on depressed mood.

The majority of the individuals in our sample were women (57.2%) (Table 2). About 40% were married or cohabiting. The percentage of individuals belonging to the six main ethnic origin groups in our study, i.e. Dutch, South-Asian Surinamese, African Surinamese, Ghanaian, Turkish, and Moroccan, ranged from 10% (Ghanaian) to 20% (Dutch). Of the individuals with a migration background (*n* = 17,528), most were first-generation (76%). In the study sample, 14.6% of individuals experienced depressed mood, with considerable variation across those of different ethnic origins (7% among those of Dutch ethnic origin, around 20% among those of South-Asian Surinamese, Moroccan and Turkish ethnic origin, and 9–10% among those of other Surinamese ethnic origin and those of Ghanaian ethnic origin). The percentage of those with no or elementary schooling was higher and of those with higher vocational schooling and university was lower among those with depressed mood than among those without depressed mood. In addition, among those with depressed mood the percentage of those experiencing the precariousness indicators was higher (Table 2). Supplementary Table 1 presents Table 2 with row percentages instead of column percentages.

**Fig. 1:**
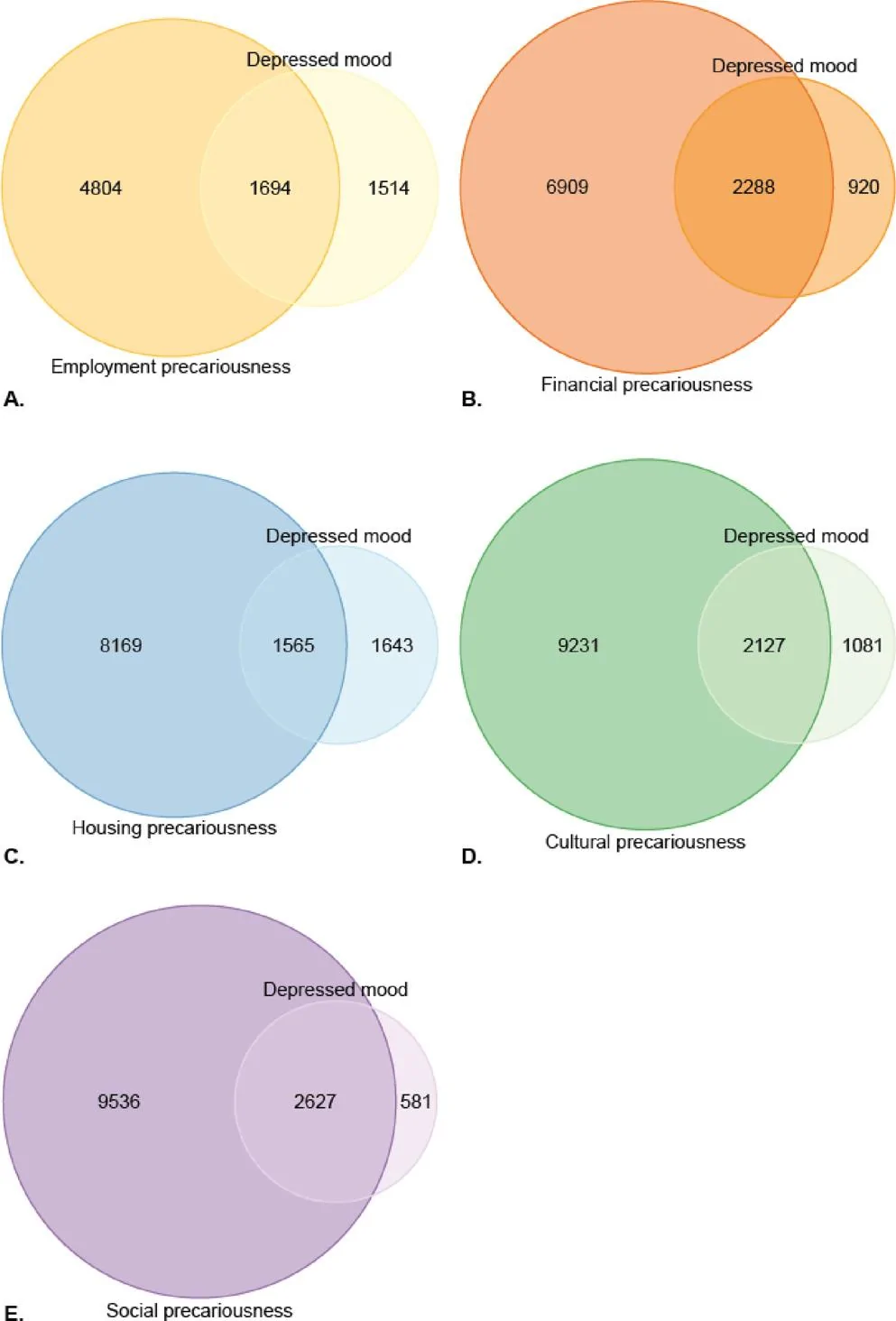
Venn diagrams of the overlap in the number of individuals experiencing depressed mood and precariousness in the different dimensions among a total of 22,039 individuals: (**A**) Employment (*n* = 8,012 individuals included in the diagram), (**B**) Financial (*n* = 10,117), (**C**) Housing (*n* = 11,377), (**D**) Cultural (*n* = 12,439), and (**E**) Social dimension (*n* = 12,744). Each of the Venn diagrams is scaled, but the different Venn diagrams are not scaled relative to each other

Figure 1 shows the Venn diagrams of the overlap in the experience of precariousness in the different dimensions and the experience of depressed mood. Figure 1E shows that over 80% (2,627 out of 3,208) of the individuals with depressed mood experience precariousness in the social dimension, whereas this is 51% among those without depressed mood. In the financial and cultural dimension, the percentages are around 70% for those with depressed mood vs. 37% and 49%, respectively, among those without depressed mood (Fig. 1A and C). Finally, around 50% of those experiencing depressed mood experience precariousness in the employment or the housing dimension vs. 26% and 43%, respectively, of those without depressed mood. Absolute and relative differences in the percentage of those with depressed mood and those without who experience precariousness in the different dimensions are thus biggest for the financial (34%, 1.9 times), employment (27%, 2.0 times), and social (31%, 1.6 times) dimensions. The differences are smaller for the cultural dimension (17%, 1.3 times) and nearly absent for the housing dimension (6%, 1.1 times).

**Fig. 2:**
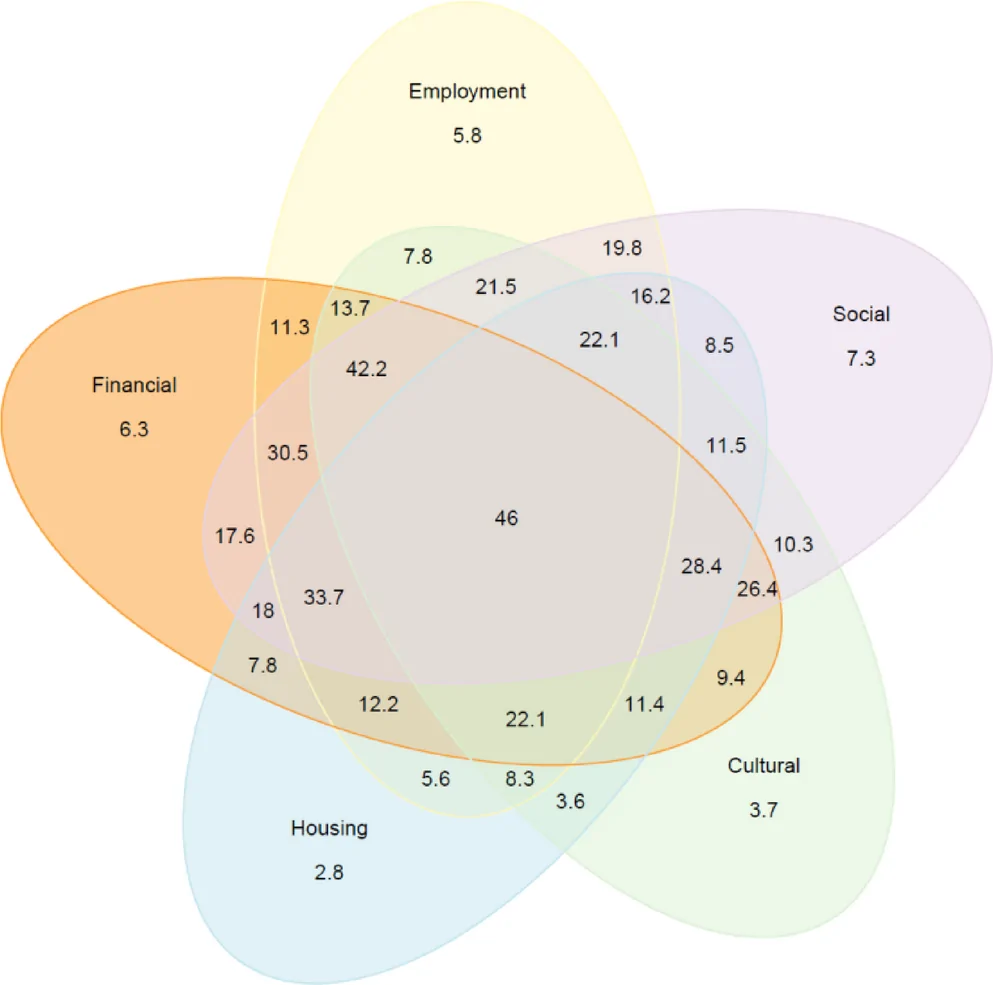
The percentage of individuals with depressed mood out of those experiencing a certain combination of precariousness dimensions (*n* = 19,785 individuals in the sample experience precariousness in at least one of the five dimensions)

Nearly 90% of the sample experiences precariousness according to at least one indicator. As individuals can experience precariousness on one indicator when they work less than 12 h per week, experience a break-up, or when they live in an area with a high percentage of rental properties, this high percentage is not surprising. Figure 2 shows that among those experiencing precariousness in just one dimension, the percentage with depressed mood is actually lower than the prevalence of depression in the study sample (14.6%). It ranges from 2.8% (housing dimension) to 7.3% (social dimension). Among those who experience precariousness in 4 dimensions, the percentage of those with depressed mood is consistently higher than 14.6% (22.1% to 42.2% across different combinations of the precariousness dimensions). Here, the percentage is lowest in the combination with all but the social dimension or the financial dimension and highest in the combination with all but the housing dimension. Of those who experience precariousness in all five dimensions, nearly half (46%) experience depressed mood.

**Fig. 3:**
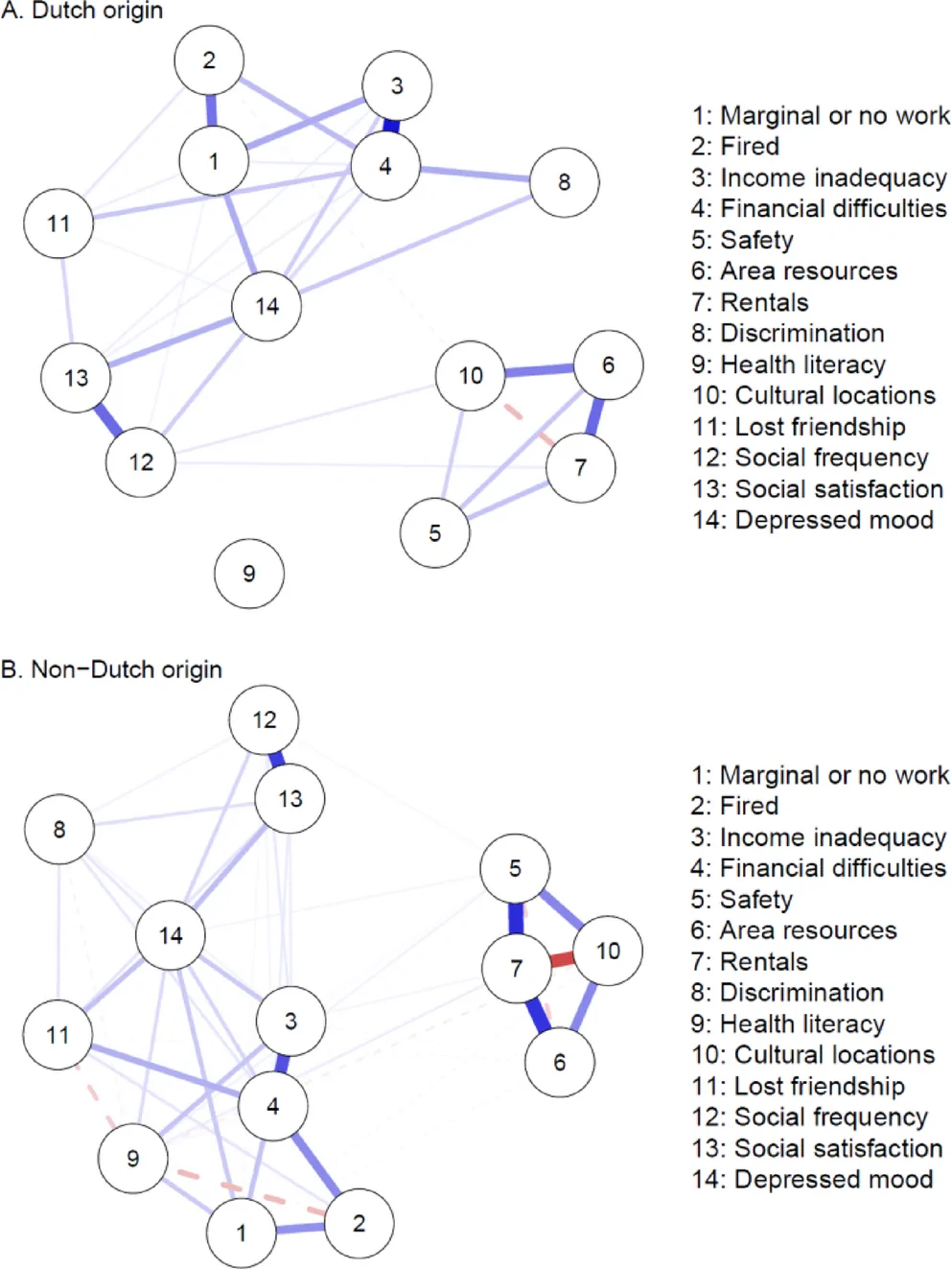
Ising model of the precariousness indicators and depressed mood for those of (**a**) Dutch ethnic origin (*n* = 4,511) and (**b**) non-Dutch ethnic origin (*n* = 17,528). Edge thickness and color intensity is in line with the strength of the edge weight and can be compared between the two models (bootstrapped confidence intervals of the edge weights can be found in Supplemental Figs. 1 and 2). Blue edges (solid) represent positive and red edges (dashed) represent negative associations. Note: the association between node 7 and 10 in the non-Dutch ethnic origin group is negative, but due to the proximity of the variables it does not appear to be dashed

In the stratified network models including all precariousness indicators and depressed mood, depressed mood is associated with many indicators of precariousness. Results for those with and without a migration background are very similar. Depressed mood is associated with 6 precariousness indicators (out of 13) among those of Dutch ethnic origin and 8 precariousness indicators among those of non-Dutch ethnic origin. Among those of Dutch ethnic origin, depressed mood is associated with marginal work or unemployment, social satisfaction, social frequency, income inadequacy, discrimination, and financial difficulties (Fig. 3A), although the confidence intervals of the associations with both discrimination and financial difficulties are wide (Supplemental Fig. 1). The confidence intervals for these associations are much smaller in the network for those of non-Dutch ethnic origin, although the association with discrimination is relatively weak (Supplemental Fig. 2). For those of non-Dutch ethnic origin, associations are also identified between depressed mood and lost friendship and health literacy (Fig. 3B). As there may be differences in the associations between the various groups of non-Dutch ethnic origin, we performed post-hoc analyses exploring the network models for each ethnic origin group with a migration background. These show that the relatively weak associations of discrimination, health literacy and social frequency with depressed mood are not present in all non-Dutch ethnic origin groups. In addition, the association with financial difficulties is weak with relatively wide confidence intervals in some of the groups. Finally, among those of Ghanaian ethnic origin, the confidence intervals around the associations with social satisfaction and income inadequacy are wide and there seems to be a weak association with being fired or becoming unemployed, although the confidence interval of this association is also wide.

As a test of robustness of our findings, we performed the second step of the analysis for general mental health instead of depressed mood. Although a larger percentage experienced low general mental health (*n* = 5,148, 23.5%) than depressed mood, findings regarding associations between indicators of precariousness and general mental health were very similar (Supplemental Fig. 3). The only noteworthy differences when using general mental health were that among those with Dutch ethnic origin, general mental health was not associated with discrimination, but was associated with lost friendship. Therefore, the associations between precariousness indicators and poor mental health for those of non-Dutch ethnic origin and those of Dutch ethnic origin are even more similar than in the main analysis.

**Table 2:** Demographic factors and precariousness indicators according to depressed mood and overall

	Depressed mood		
	No	Yes	Overall
	(*N* = 18,831)	(*N* = 3,208)	(*N* = 22,039)
**Ethnic origin**			
Dutch	4190 (22.3%)	321 (10.0%)	4511 (20.5%)
South-Asian Surinamese	2535 (13.5%)	572 (17.8%)	3107 (14.1%)
African Surinamese	3703 (19.7%)	424 (13.2%)	4127 (18.7%)
Javanese Surinamese	212 (1.1%)	20 (0.6%)	232 (1.1%)
Surinamese (other/unknown)	229 (1.2%)	26 (0.8%)	255 (1.2%)
Ghanaian	2014 (10.7%)	197 (6.1%)	2211 (10.0%)
Turkish	2865 (15.2%)	851 (26.5%)	3716 (16.9%)
Moroccan	3083 (16.4%)	797 (24.8%)	3880 (17.6%)
**Sex**			
Men	8356 (44.4%)	1078 (33.6%)	9434 (42.8%)
Women	10,475 (55.6%)	2130 (66.4%)	12,605 (57.2%)
**Age**, mean (SD)	44.0 (13.5)	43.1 (12.8)	43.9 (13.4)
**Education**			
No or elementary schooling	2944 (15.6%)	816 (25.4%)	3760 (17.1%)
Lower vocational or secondary schooling	4898 (26.0%)	911 (28.4%)	5809 (26.4%)
Intermediate vocational/higher secondary schooling	5578 (29.6%)	978 (30.5%)	6556 (29.7%)
Higher vocational schooling or university	5340 (28.4%)	490 (15.3%)	5830 (26.5%)
Missing	71 (0.4%)	13 (0.4%)	84 (0.4%)
**Marital status**			
Married/registered partnership	7336 (39.0%)	1126 (35.1%)	8462 (38.4%)
Cohabiting	2196 (11.7%)	211 (6.6%)	2407 (10.9%)
Unmarried	6530 (34.7%)	1113 (34.7%)	7643 (34.7%)
Divorced	2331 (12.4%)	667 (20.8%)	2998 (13.6%)
Widowed	340 (1.8%)	80 (2.5%)	420 (1.9%)
Missing	98 (0.5%)	11 (0.3%)	109 (0.5%)
**Occupational level**			
Elementary	2426 (12.9%)	511 (15.9%)	2937 (13.3%)
Lower	4823 (25.6%)	967 (30.1%)	5790 (26.3%)
Intermediate	4360 (23.2%)	705 (22.0%)	5065 (23.0%)
Higher	3384 (18.0%)	349 (10.9%)	3733 (16.9%)
Academic	1294 (6.9%)	78 (2.4%)	1372 (6.2%)
Missing	2544 (13.5%)	598 (18.6%)	3142 (14.3%)
**Migration generation**			
First generation	11,183 (59.4%)	2208 (68.8%)	13,391 (60.8%)
Non-migrants	4190 (22.3%)	321 (10.0%)	4511 (20.5%)
Second generation	3458 (18.4%)	679 (21.2%)	4137 (18.8%)
**Precariousness indicators**			
Marginal work or unemployment	3922 (20.8%)	1490 (46.4%)	5412 (24.6%)
Fired or became unemployed (< 12 months)	1885 (10.0%)	600 (18.7%)	2485 (11.3%)
Income inadequacy	6311 (33.5%)	2138 (66.6%)	8449 (38.3%)
Financial difficulties (< 12 months)	2909 (15.4%)	1382 (43.1%)	4291 (19.5%)
Low safety neighborhood	4328 (23.0%)	905 (28.2%)	5233 (23.7%)
Low area resources	4074 (21.6%)	756 (23.6%)	4830 (21.9%)
High percentage rentals	3753 (19.9%)	749 (23.3%)	4502 (20.4%)
Discrimination	4218 (22.4%)	1283 (40.0%)	5501 (25.0%)
Low health literacy	2427 (12.9%)	772 (24.1%)	3199 (14.5%)
Low number of cultural locations	4586 (24.4%)	830 (25.9%)	5416 (24.6%)
Break-up or lost friendship	3625 (19.3%)	1324 (41.3%)	4949 (22.5%)
Low frequency of social support	5756 (30.6%)	1941 (60.5%)	7697 (34.9%)
Low satisfaction with social support	5428 (28.8%)	1956 (61.0%)	7384 (33.5%)
**Precariousness score**, median [range]	2 [0,10]	5 [0,10]	3 [0,10]

The reported percentages are referring to the share of the column totals

## Discussion

In this study, we examined the association between experiences of precariousness and depressed mood while accounting for, and making explicit, the underlying associations between experiences of precariousness. We found that individuals with and without depressed mood differ markedly in their experiences of precariousness. Depressed mood is related to many different aspects of precariousness. However, differences between those with and without depressed mood in the exposure to precarious neighborhood characteristics are small. Depressed mood is associated with experiences of precariousness in employment (primarily the experience of marginal or unemployment), finances and social life, and in groups with non-Dutch ethnic origin also with health literacy. Further, among those who experience precariousness in many different dimensions of life, the percentage of individuals with depressed mood is substantially higher, specifically when experiences in the social and financial dimension of life are included. Overall, depressed mood is, thus, highly intertwined with precariousness. In addition, patterns in the associations between experiences of precariousness and depressed mood were relatively similar for those of Dutch and non-Dutch ethnic origin, highlighting precariousness as a potential shared mechanism for depressed mood in these population groups.

Precariousness arose as a concept in the French scientific literature in the late 1970s to describe families living in conditions that made them vulnerable to incidents or events [36]. As such, it described families, or individuals, that were ‘living on the edge’ [1]. Insecurity, instability, and hardship were described in working conditions, pay, finances, housing, health, and social relations. Importantly, although employment was rather central to this concept, it was only one aspect of it [36]. Precariousness was suggested to increase an individual’s chances, whether real or perceived, of experiencing adversity [1, 37]. Precariousness can, thus, be regarded as a multidimensional social position where people are at a greater risk of experiencing adverse situations [38], in line with the operationalization in the current study. Over the last couple of decades, precariousness has primarily been examined in relation to employment and housing, with limited inclusion of other dimensions of life [1, 39]. However, experiences of precariousness can be manifold, will often arise in multiple dimensions of life, and collectively give rise to a state of living in insecurity and instability. It is important to emphasize that experiencing precariousness according to one indicator in the current study should thus not be regarded as experiencing a state of precariousness. An individual precarious experience is also not necessarily detrimental, which is also shown by the fact that among individuals who experienced precariousness in just one dimension – or even on just one indicator – the proportion with depressed mood was relatively low.

Financial precariousness (i.e., income inadequacy and financial difficulties), employment precariousness (i.e., marginal or unemployment), and social precariousness (i.e., low frequency of social support, low satisfaction with social support, and lost friendship or break up) were most intricately related to depressed mood. These associations were present while accounting for all other experiences, meaning that other precariousness experiences did not explain these associations. Previous studies found that precarious employment and precarious housing adversely affected self-reported health and mental health [3, 40]. Studies on precarious employment and health suggested explanatory mechanisms such as poor working conditions, feelings of injustice and powerlessness, and social and material deprivation [4, 7, 41]. A study on how financial precariousness was related to wellbeing among international students in Australia suggested that financial precarity could be related to the need to work and housing affordability, and to physical and psychological well-being through shortage of time, worries, food insecurity, and having difficulties making friends [6]. The authors also highlighted how these experiences can create vicious circles, as wellbeing may not only be caused by, but also may cause financial precariousness. Although our study population is different, we expect similar mechanisms and vicious circles to be at work in this population, and our results suggest that there is a crucial role for financial precariousness.

The network analysis offers valuable insights into potential pathways linking experiences of precariousness and depressed mood. For instance, a lack of social support could lead to depressed mood, unemployment, and financial struggles, but conversely, unemployment and financial difficulties could contribute to depressed mood and a subsequent decline in social support. Additionally, it is possible that a depressed mood could drive both unemployment and financial hardships, as well as a reduction in social support. Given that depressed mood is known to influence social life, it is likely that these factors form a vicious circle, where depressed mood both contributes to and is exacerbated by unemployment and financial struggles, while also leading to a lack of social support. Furthermore, negative mood states may contribute to cognitive biases, which can lead individuals to perceive social and financial difficulties as more severe or insurmountable than they objectively are [42, 43]. This negative cognitive appraisal can reinforce maladaptive coping mechanisms, reduce problem-solving capacity, and diminish motivation to seek support, thereby perpetuating the cycle of precariousness and depression [43, 44]. Such self-reinforcing feedback loops, in which psychological distress exacerbates the perception of adversity, underscore the importance of addressing both adverse conditions and internal cognitive-emotional processes in interventions aimed at breaking this cycle [45].

While the main limitation of this study is its cross-sectional nature, the main strength is the incorporation of many indicators of precariousness in different dimensions of life which allowed us to shed light on the links between all these precariousness experiences and depressed mood in a network analysis. These models are undirected and do not imply causality, but they can highlight potential paths and clusters of interconnected experiences that may warrant further investigation. They are a useful departure point for examining complex concepts as they enable the identification of potential leverage points for intervention that require further investigation [46, 47], though we emphasize caution in interpreting centrality metrics in order to identify these leverage points, in line with recent methodological critiques [48]. It should be kept in mind, though, that omitted variables could influence identified associations [49]. The included indicators are secondary data that fitted the concept of precariousness. For the housing dimension, we were only able to include neighborhood-level indicators of precariousness, rather than individual-level indicators, and we had limited information on precariousness in the cultural dimension. Though we were able to include a wide array of dimensions and indicators, these are not fixed or exhaustive. As the relevant indicators to include will depend on the population of the study and the day, age and context in which the study is performed, creating a comprehensive list of the indicators to include is, in our view, not realistic. Another strength of this study is the inclusion of groups with various ethnic origins and the examination of the associations between precariousness experiences and depressed mood in these different groups.

The fact that hardly any associations were identified between objectively measured neighborhood characteristics and depressed mood is interesting, as it seems to indicate that these characteristics are not very important for the experience of depressed mood. This contrasts with the expectation that, for example, an unsafe neighborhood has impact on mental health [50]. However, these findings should not be overinterpreted. Weaker associations between indicators measured at the neighborhood rather than the individual level when assessing the association with depressed mood are actually to be expected. Further, the experiences of precariousness measured at the individual level are measured using the same questionnaire, which could inflate differences between the experiences at the individual level and experiences at the neighborhood level due to common method bias. A final limitation is that generalizability of the findings of this study may be limited as precariousness is highly context-dependent, where context refers to the cultural, political, economic, and social background in which precariousness arises, which will be influenced by geographical location (e.g., country, region, degree of urbanicity) as well as by time [4, 51]. Nonetheless, we expect the findings to translate to other countries with similar social security systems and social contexts.

### Implications

Currently, the cost of living in European countries is rising rapidly, putting more people at risk for financial difficulties, uncertain living conditions, and health issues [52]. Consequently, there is a risk of a rise in the number of individuals living in precarious circumstances and consequent health inequalities. Experiences of precariousness arise in and from a certain political and structural context. Adequate social security is suggested to protect against experiences of precariousness [1, 36] and changes in social security have substantial implications for the insecurity and instability experienced in the different, but related, dimensions of life and for mental health [5, 9, 53]. For example, one study found that in a period where benefit levels decreased and eligibility criteria became more strict, mental distress in women increased most among those outside the labor market, which could indicate that their financial difficulties and stress went up [54]. Another study found that reductions in housing benefits led to an increase in depressive symptoms for individuals with a low income renting in the private sector [11]. Austerity has also been shown to have a deteriorating effect on health, mostly for those whose lives are already insecure, such as those precariously employed, in precarious housing, or with pre-existing health problems [55]. The current study suggests that a very diverse array of precariousness experiences in many different dimensions of life are related to depressed mood. In line with previous studies, it therefore suggests that reducing health inequalities requires a concerted effort directed at social, environmental, and economic factors [56]. It further underscores that research into possible targets of interventions and policies must happen in an integrated fashion [52]. Finally, it emphasizes the need to focus on prevention of cascading effects of insecurity and instability from one dimension of life to another. Based on the results of earlier studies, it is possible that social security has a role to play in reducing these cascading effects.

## Conclusion

This integrated explorative study underscores the multi-faceted nature of precariousness and its multi-faceted associations with depressed mood and allows for the identification of potential leverage points for intervention. The interrelation between these experiences and phenomena suggests a likely cascading effect of experiences of precariousness and depressed mood. Although the precise interactions between these aspects are unclear, better protection against income inadequacy and financial difficulties could potentially break the vicious circles between these factors and depressed mood. However, concerted efforts addressing several aspects are likely required, as precariousness in the employment and social dimension also seem important for the experience of depressed mood.

## Supplementary Information

Below is the link to the electronic supplementary material.

Supplementary Material 1

## Data Availability

The HELIUS data are owned by the Amsterdam UMC, location AMC in Amsterdam, the Netherlands. Any researcher can request the data by submitting a proposal to the HELIUS Executive Board as outlined at http://www.heliusstudy.nl/en/researchers/collaboration, by email: heliuscoordinator@amsterdamumc.nl. The HELIUS Executive Board will check proposals for compatibility with the general objectives, ethical approvals and informed consent forms of the HELIUS study. There are no other restrictions to obtaining the data and all data requests will be processed in the same manner.
