# Supplementary material for: Precariousness and depressed mood: a network analysis in the multi-ethnic HELIUS study
Source: Soc Psychiatry Psychiatr Epidemiol. 2026 Mar 13;61(10):2235–46. doi: 10.1007/s00127-026-03072-w (PMC13585958; doi:10.1007/s00127-026-03072-w)
Supplement: Supplementary file 1 [file 127_2026_3072_MOESM1_ESM.pdf]

Article title: Precariousness and depressed mood: a network analysis in the multi-ethnic HELIUS cohort study

Journal name: Social Psychiatry and Psychiatric Epidemiology

Author names: Leonie K. Elsenburg [a,b], Karien Stronks [a], Henrike Galenkamp [a], Jeroen Lakerveld [c,d], Anja Lok [e,b], Kyuri Park [f], Vítor V. Vasconcelos [f,b,g], Mary Nicolaou [a]

[a] Department of Public and Occupational Health, Amsterdam Public Health Research Institute, Amsterdam UMC, University of Amsterdam, Amsterdam, Netherlands.
[b] The Centre for Urban Mental Health, University of Amsterdam, Amsterdam, Netherlands
[c] Department of Epidemiology & Data Science, Amsterdam Public Health Research Institute, Amsterdam UMC, Vrije Universiteit Amsterdam, Amsterdam, Netherlands.
[d] Upstream Team, Amsterdam UMC, Vrije Universiteit Amsterdam, Amsterdam, Netherlands
[e] Department of Psychiatry, Amsterdam University Medical Centers, Location AMC, Meibergdreef 5, 1105, AZ, Amsterdam, the Netherlands
[f] Computational Science Lab, Informatics Institute, University of Amsterdam, Amsterdam, Netherlands
[g] POLDER center, Institute for Advanced Study, University of Amsterdam, Amsterdam, Netherlands

Corresponding author: Leonie K. Elsenburg, PhD

Supplemental Table 1. Demographic and precariousness indicators according to depressed mood

	Depressed mood			
	No		Yes	
	n	%	n	%
	18831	85.4	3208	14.6
**Ethnic origin**				
Dutch	4190	92.9	321	7.1
South-Asian Surinamese	2535	81.6	572	18.4
African Surinamese	3703	89.7	424	10.3
Javanese Surinamese	212	91.4	20	8.6
Surinamese (other/unknown)	229	89.8	26	10.2
Ghanaian	2014	91.1	197	8.9
Turkish	2865	77.1	851	22.9
Moroccan	3083	79.5	797	20.5
**Sex**				
Men	8356	88.6	1078	11.4
Women	10475	83.1	2130	16.9
**Education**				
No or elementary schooling	2944	78.3	816	21.7
Lower vocational or secondary schooling	4898	84.3	911	15.7
Intermediate vocational/higher secondary schooling	5578	85.1	978	14.9
Higher vocational schooling or university	5340	91.6	490	8.4
Missing	71	84.5	13	15.5
**Marital status**				
Married/registered partnership	7336	86.7	1126	13.3
Cohabiting	2196	91.2	211	8.8
Unmarried	6530	85.4	1113	14.6
Divorced	2331	77.8	667	22.2
Widowed	340	81.0	80	19.0
Missing	98	89.9	11	10.1
**Occupational level**				
Elementary	2426	82.6	511	17.4
Lower	4823	83.3	967	16.7
Intermediate	4360	86.1	705	13.9
Higher	3384	90.7	349	9.3
Academic	1294	94.3	78	5.7
Missing	2544	81.0	598	19.0
**Migration generation**				
First generation	11183	83.5	2208	16.5
Non-migrants	4190	92.9	321	7.1
Second generation	3458	83.6	679	16.4
**Precariousness indicators**				
Marginal work or unemployment	3922	72.5	1490	27.5
Fired (<12 months)	1885	75.9	600	24.1
Income inadequacy	6311	74.7	2138	25.3
Financial difficulties (<12 months)	2909	67.8	1382	32.2
Unsafe neighborhood	4328	82.7	905	17.3
Low area resources	4074	84.3	756	15.7
High percentage rentals	3753	83.4	749	16.6
Discrimination	4218	76.7	1283	23.3
Low health literacy	2427	75.9	772	24.1
Low number of cultural locations	4586	84.7	830	15.3
Break-up or lost friendship	3625	73.2	1324	26.8
Low frequency of social support	5756	74.8	1941	25.2
Low satisfaction with social support	5428	73.5	1956	26.5

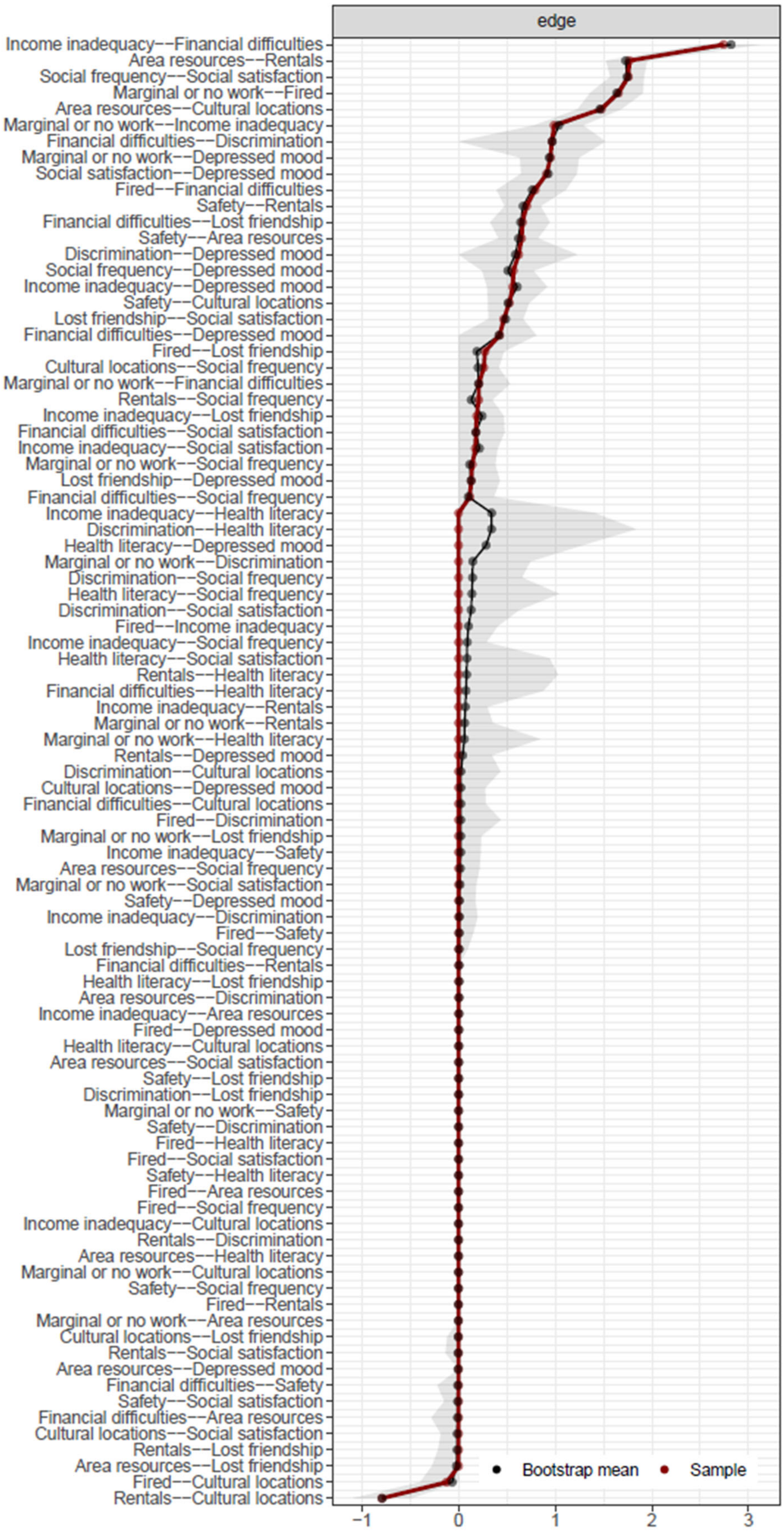

Supplemental Figure 1. Bootstrapped confidence intervals of the edge weights for the Dutch ethnic group (n=4,511)

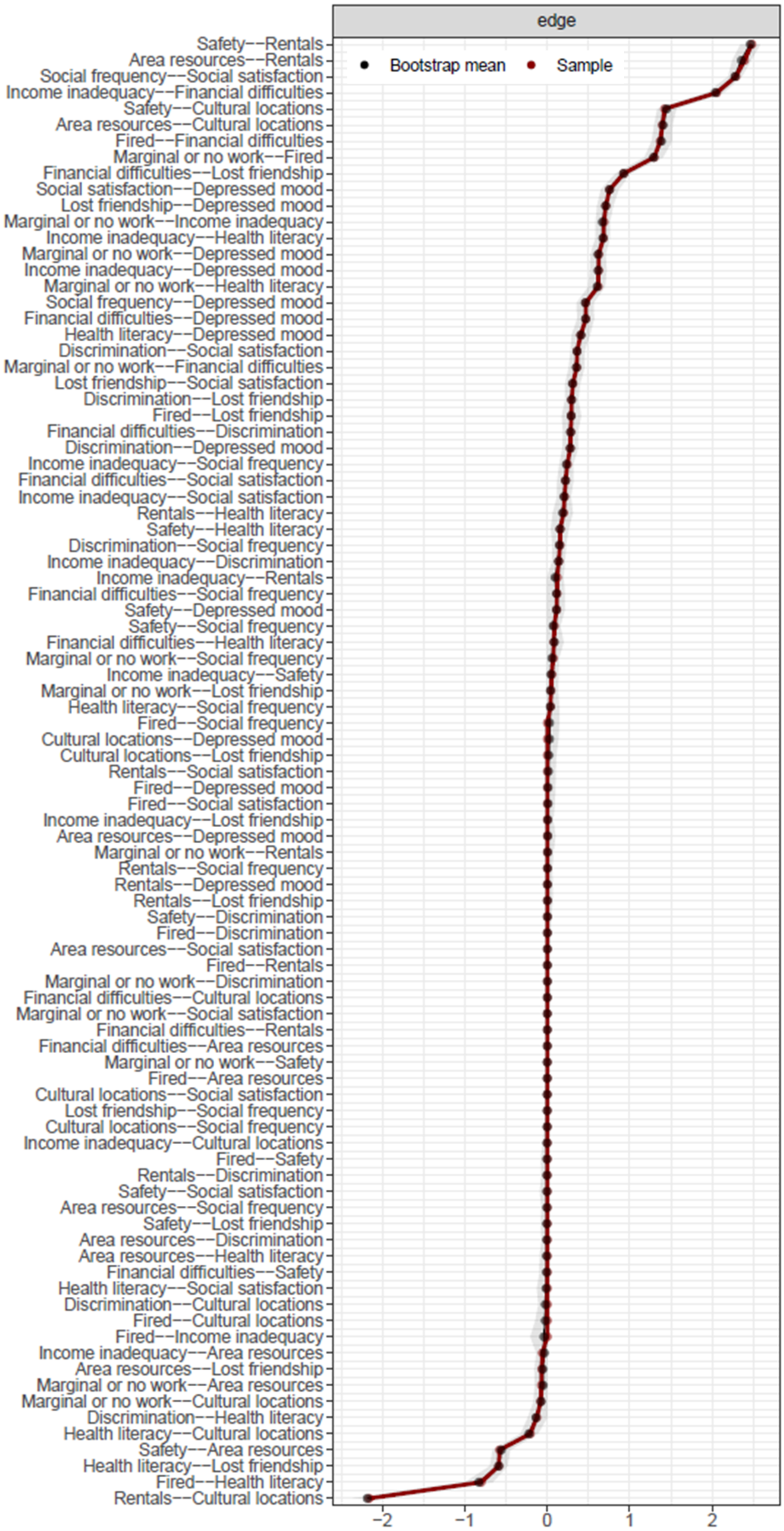

Supplemental Figure 2. Bootstrapped confidence intervals of the edge weights for the non-Dutch ethnic group (n=17,528)

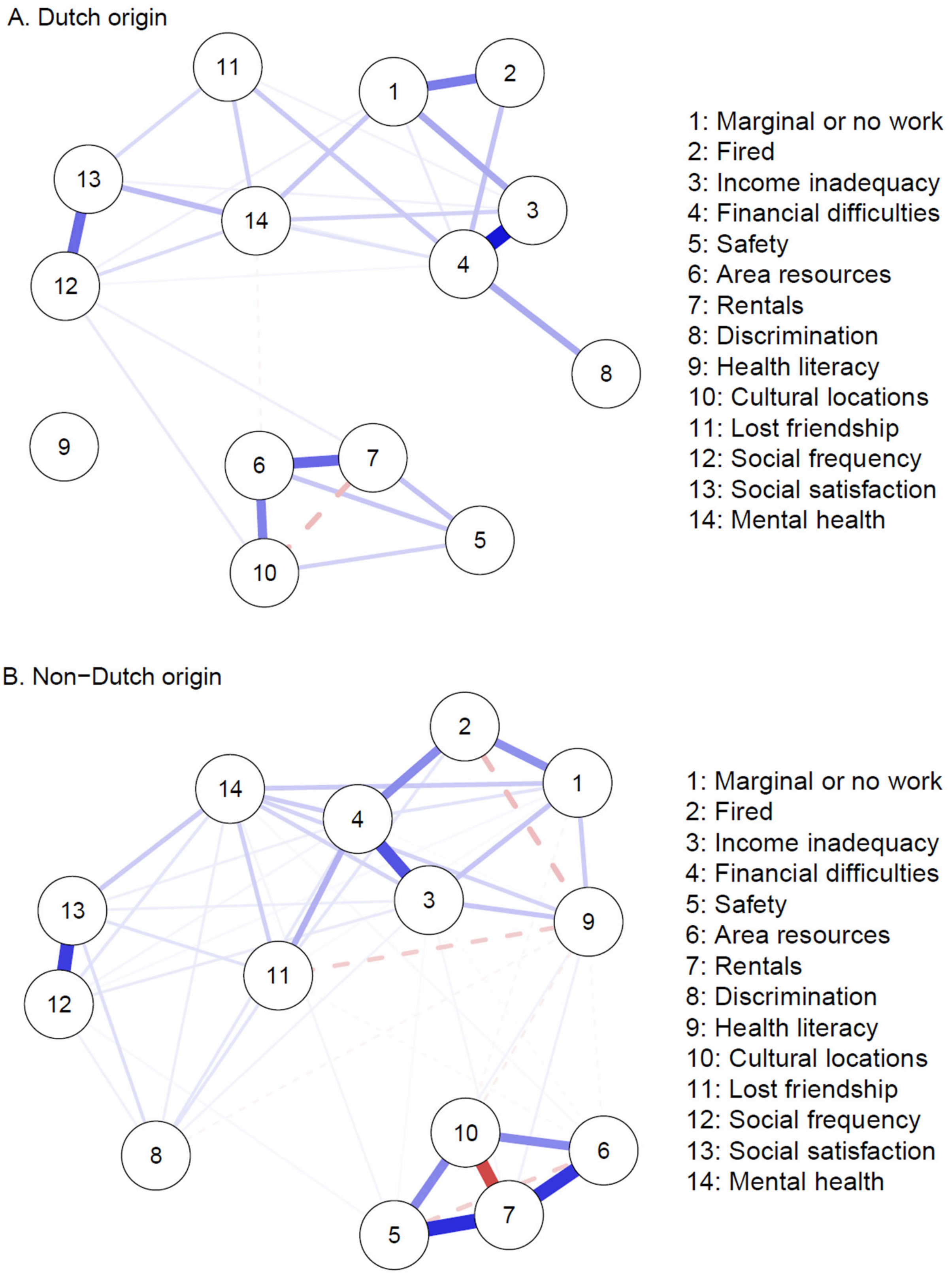

Supplemental Figure 3. Ising model of the precariousness indicators and low general mental health for (a) those with Dutch ethnic origin (n=4,505) and (b) for those with non-Dutch ethnic origin (n=17,443). Edge thickness and color intensity is in line with the strength of the edge weight and can be compared between the models. Blue edges (solid) represent positive and red edges (dashed) represent negative associations. Note: the association between node 7 and 10 in the non-Dutch ethnic group is negative, but due to the proximity of the variables it does not appear to be dashed.
